# The Characteristic Fragrant Sesquiterpenes and 2-(2-Phenylethyl)chromones in Wild and Cultivated “Qi-Nan” Agarwood

**DOI:** 10.3390/molecules26020436

**Published:** 2021-01-15

**Authors:** Li Yang, Jin-Ling Yang, Wen-Hua Dong, Ya-Li Wang, Jun Zeng, Jing-Zhe Yuan, Hao Wang, Wen-Li Mei, Hao-Fu Dai

**Affiliations:** 1 Hainan Engineering Research Center of Agarwood, Institute of Tropical Bioscience and Biotechnology, Chinese Academy of Tropical Agricultural Sciences, Haikou 571101, China; yangli@itbb.org.cn (L.Y.); jinlyang@126.com (J.-L.Y.); dongwenhua@itbb.org.cn (W.-H.D.); wyl200881@163.com (Y.-L.W.); zengjun@itbb.org.cn (J.Z.); yuanjingzhenpc@126.com (J.-Z.Y.); wanghao@itbb.org.cn (H.W.); 2 Hainan Key Laboratory for Research and Development of Natural Products from Li Folk Medicine, Institute of Bioscience and Biotechnology, Chinese Academy of Agricultural Sciences, Haikou 571101, China; 3 Hainan Institute for Tropical Agricultural Resources, Chinese Academy of Agricultural Sciences, Haikou 571101, China

**Keywords:** cultivated “Qi-Nan” agarwood, wild harvested “Qi-Nan” agarwood, GC-MS, 2-(2-phenylethyl)chromone, sesquiterpene, fragrant component

## Abstract

Recently, cultivated “Qi-Nan” (CQN) agarwood has emerged as a new high-quality agarwood in the agarwood market owing to its similar characteristics, such as high content of resin and richness in two 2-(2-phenylethyl)chromone derivatives, 2-(2-phenylethyl)chromone (**59**) and 2-[2-(4-methoxyphenyl)ethyl]chromone (**60**), to the wild harvested “Qi-Nan” (WQN) agarwood. In this study, we compared the chemical constituents and fragrant components of two types of WQN agarwood from *A. agallocha* Roxb. and *A. sinensis*, respectively, with CQN agarwood and ordinary agarwood varieties. Additionally, we analyzed different samples of WQN agarwood and CQN agarwood by GC-MS, which revealed several noteworthy differences between WQN and CQN agarwood. The chemical diversity of WQN was greater than that of CQN agarwood. The content of (**59**) and (**60**) was higher in CQN agarwood than in WQN agarwood. For the sesquiterpenes, the richness and diversity of sesquiterpenes in WQN agarwood, particularly guaiane and agarofuran sesquiterpenes, were higher than those in CQN. Moreover, guaiane-furans sesquiterpenes were only detected by GC-MS in WQN agarwood of *A. sinensis* and could be a chemical marker for the WQN agarwood of *A. sinensis*. In addition, we summarized the odor descriptions of the constituents and established the correlation of scents and chemical constituents in the agarwood.

## 1. Introduction

Agarwood, also called eaglewood, gaharu, jinko, aloeswood, pokok karas, chen xiang, kalambak, or oud in different countries, is a resinous heartwood from the *Aquilaria* and *Gyrinops* species of the Thymelaeceae family and is formed after physical wounding of the stem or infection by pathogens [1,2,3]. As a traditional medicine, agarwood was used for the treatment of various diseases, including rheumatism, arthritis, body pain, asthma, gout, and also acted as a stimulant as well as sedative, analgesic, and carminative agent [4]. Moreover, agarwood is highly valued for its usage in incense and perfume due to its special and pleasant fragrance [5]. “Qi-Nan”, also named Kanankoh, Kyara, or Chi-Nan in different cultures, is commonly considered as the highest quality agarwood in the market, mainly owing to its mysterious and elegant scent, resinoid content, color, and morphological characteristics which are distinct from those of ordinary agarwood [6]. Due to its scarcity and preciousness, “Qi-Nan” is highly appreciated and its price is hundreds or even thousands of times higher than that of ordinary agarwood [5]. The huge economic value and the exhausted wild resource of agarwood have promoted the rapid emergence of the cultivated “Qi-Nan” in recent years. *A. sinensis* “Reke2”, for example, is a high-quality cultivated “Qi-Nan” propagated by grafting with the ordinary germplasm of *A. sinensis*, and can produce agarwood with higher content of resin than that of ordinary *A. sinensis* trees after artificial induction [7]. However, there is little information concerning the difference between the wild and cultivated “Qi-Nan” agarwood varieties in terms of the chemical constituents and aroma components. Li analyzed the incense smoke of cultivated grafting “Qi-Nan” agarwood using thermogravimetric fourier transform infrared spectroscopy (TG-FTIR) and headspace gas chromatography–mass spectrometry (HSGC–MS), and observed a large difference in the main components, including sesquiterpenes and aromatic compounds, between cultivated grafting “Qi-Nan” and wild “Qi-Nan” agarwood. However, this study did not determine the specific constituents, especially the 2-(2-phenylethyl)chromone derivatives, responsible for the differences [8].

**Figure 1:**
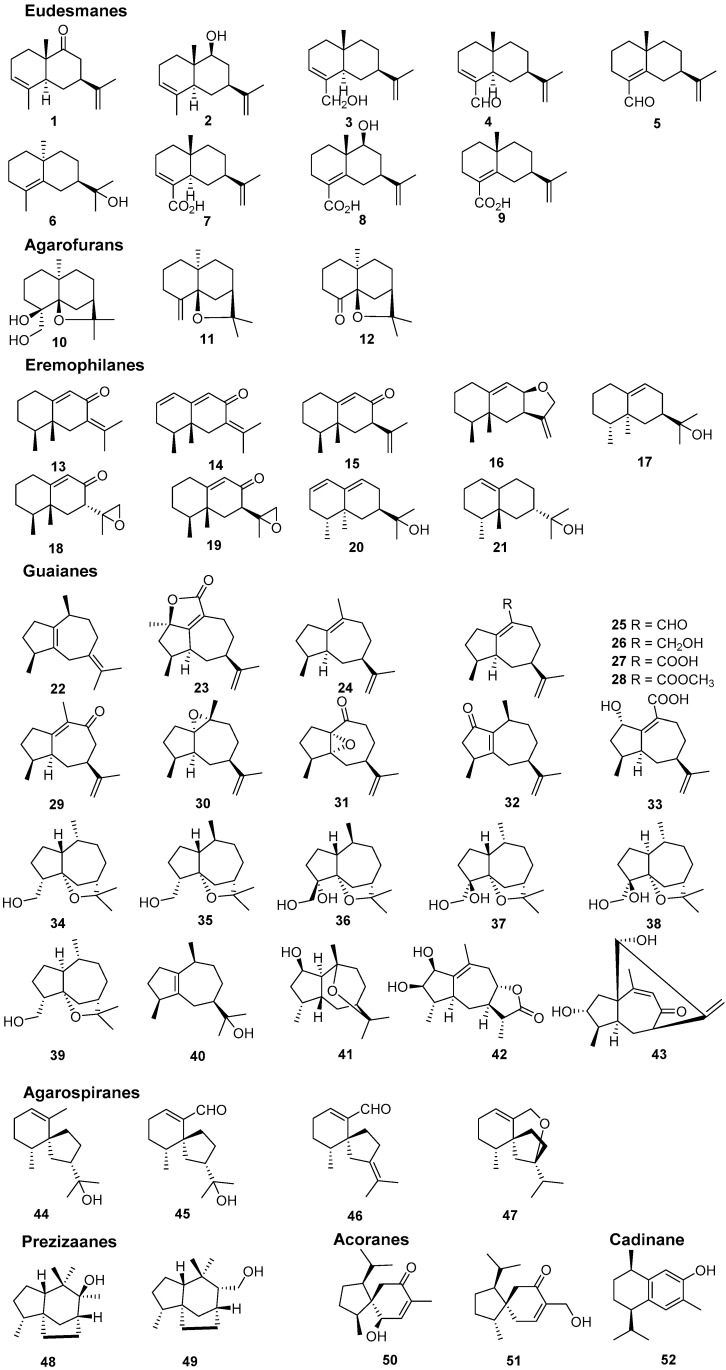
Structures of sesquiterpenes identified in “Qi-Nan” agarwood from *A. agallocha* and *A. sinensis* together with odor sesquiterpenes in agarwood.

**Figure 2:**
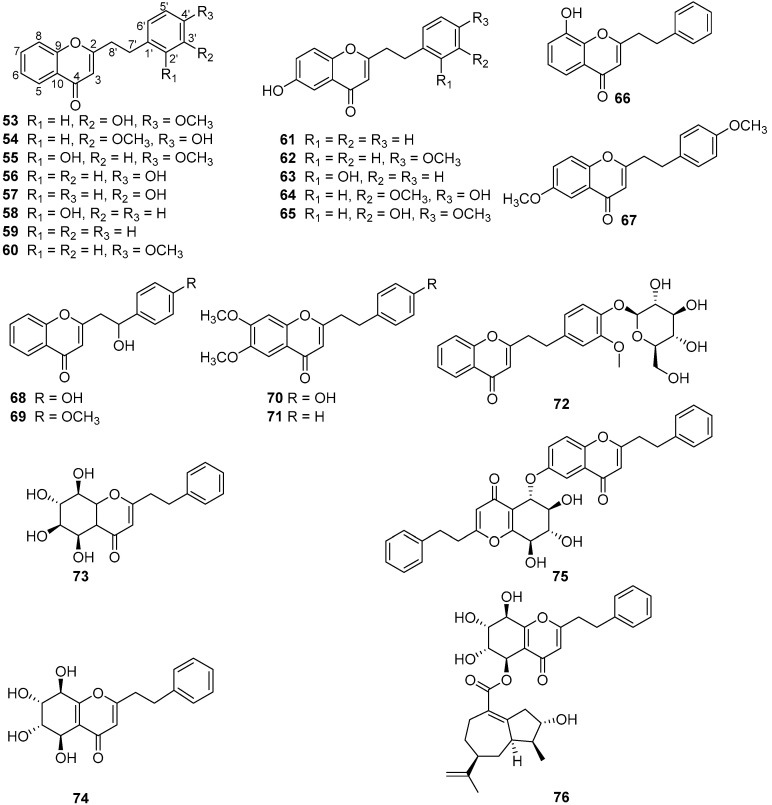
Structures of 2-(2-phenylethyl)chromones identified in “Qi-Nan” agarwood from *A. agallocha* and *A. sinensis*.

The quality of “Qi-Nan” agarwood was often assessed by the traditional grading indexes, such as physical sense, water sinking condition, color, scent/aroma, and morphology [9]. It seems that these indexes were too subjective for agarwood quality grading, and more objective grading parameters—including chemical constituents—are urgently needed. Up to now, the chemical constituents of only two wild “Qi-Nan” agarwood types, originating from *A. agallocha* Roxb. [10,11,12,13] and *A. sinensis* [14,15,16,17,18,19,20,21], respectively, have been investigated. Sesquiterpenes and 2-(2-phenylethyl)chromones were reported as their main constituents. Guaianes, eudesmanes, and eremophilanes are the three main types of sesquiterpenes found in the two wild “Qi-Nan” agarwood varieties, together with several agarofurans, agarospiranes, acoranes, cadinanes (Figure 1) [22,23]. In particular, guaianes show great structural diversity and might be used as markers for different kinds of agarwood. Most of the sesquiterpenes contribute to the special odor and to the gorgeous and elegant character of “Qi-Nan” agarwood. As for 2-(2-phenylethyl)chromones, 23 of them, except for 6-methoxy-2-[2-(4-methoxyphenyl)ethyl]chromone (**67**), have been isolated from the Chinese agarwood “Lv Qi-Nan” of *A. sinensis*, while only three—2-(2-phenylethyl)chromone (**59**), 2-[2-(4-methoxyphenyl)ethyl]chromone (**60**), and (**67**)—have been isolated from kanankoh; a high-quality agarwood of *A. agallocha* (Figure 2). Compounds (**59**) and (**60**) are two typical 2-(2-phenylethyl)chromones without substitution of the chromone moiety, which are most abundant in high quality “Qi-Nan” agarwood, as well as frequently found in most ordinary agarwood with a low relative content [22,23]. A 2-(2-phenethyl)chromone glycoside (**72**) was obtained from the high-quality “Lv Qi-Nan” of *A. sinensis*, which is the only chromone glycoside reported from agarwood up to now [17]. 

In this paper, we analyzed 22 cultivated “Qi-Nan” agarwood and seven wild “Qi-Nan” agarwood varieties by GC-MS, and compared the chemical constituents and fragrant components of cultivated “Qi-Nan” (CQN) agarwood with those of wild harvested “Qi-Nan” (WQN) agarwood and ordinary agarwood, to provide a scientific foundation for the quality evaluation of “Qi-Nan” agarwood. 

## 2. Results and Discussion

### 2.1. GC-MS Analysis of Wild Harvested “Qi-Nan” Agarwood and Cultivated “Qi-Nan” Agarwood

Seven wild harvested “Qi-Nan” agarwood samples and 22 cultivated “Qi-Nan” agarwood samples were extracted with EtOH and Et_2_O. The resin content of agarwood is a crucial quantitative criterion for grading the quality of agarwood [24]. The average ethanol extract contents of the CQN agarwood were 46.52 ± 11.25%, which were similar to, or higher than, those of the WQN agarwood (49.85 ± 9.94%), while obviously higher than those of ordinary agarwood [25,26]. Therefore, the quality of CQN agarwood was higher than that of ordinary agarwood and very close to that of WQN agarwood. 

**Figure 3:**
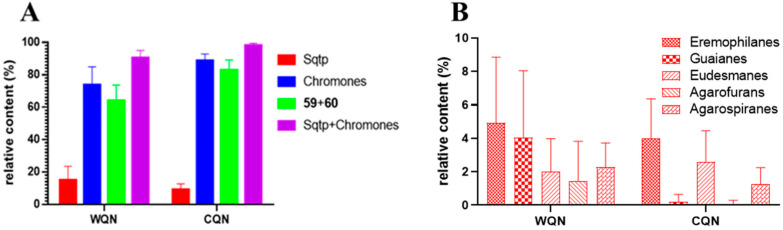
The chemical distribution in wild harvested “Qi-Nan” (WQN) agarwood and cultivated “Qi-Nan” (CQN) agarwood (Sesquiterpenoids: Sqtp). The relative contents of chromones and sesquiterpenoids in the WQN and CQN agarwood (**A**). The relative contents of five predominant types of sesquiterpenes in the WQN and CQN agarwood (**B**).

The Et_2_O extracts were analyzed by GC-MS and 56 and 40 compounds were identified in WQN agarwood and CQN agarwood, respectively, which indicated that the chemical diversity of WQN was greater than that of CQN. The main constituents in agarwood are sesquiterpenes and 2-(2-phenylethyl)chromones. As presented in Figure 3A, both of WQN and CQN showed high contents of 2-(2-phenylethyl)chromones. Especially, the relative content of the two main 2-(2-phenylethyl)chromones, 2-(2-phenylethyl)chromone (**59**) and 2-[2-(4-methoxyphenyl)ethyl]chromone (**60**), was very high (43.89–73.04% in WQN agarwood and 72.43–95.61% in CQN agarwood in this study), which was consistent with the result of our previous study [7,23] and Ishihara’s discovery [27]. Most of the CQN agarwood possessed higher contents of (**59**) and (**60**) than the levels found in WQN agarwood. Therefore, the relative content of (**59**) and (**60**) could be a reference not only for identifying and distinguishing both of WQN and CQN agarwood from ordinary agarwood, but also for discriminating the WQN from the CQN agarwood. Furthermore, detailed comparison of the structures of 2-(2-phenylethyl)chromone derivatives identified in the WQN and CQN agarwood varieties revealed that the 2-(2-phenylethyl)chromone derivatives, such as the 2-(2-phenylethyl)chromones numbered (**46**) and (**48**–**56**) in Table S4, were substituted with the hydroxy and/or methoxy on their chromone units in the WQN agarwood samples WQN1–4, while no substituents were observed on the chromone moiety of 2-(2-phenylethyl)chromone derivatives numbered (**33**–**40**) in Tables S2 and S3 in the CQN agarwood samples and WQN agarwood samples WQN5–7. This remarkable structural difference in 2-(2-phenylethyl)chromone derivatives could be sufficient evidence to determine the WQN agarwood, but is not a necessary one.

As for sesquiterpenes, their richness in WQN agarwood was higher in contrast with the CQN agarwood, as illustrated in Figure 3A. Additionally, Figure 3B provided the detailed distribution of five predominant types of sesquiterpenes, including eudesmanes, guaianes, eremophilanes, agarofurans, and agarospiranes, in the WQN and CQN agarwood varieties. Obviously, the contents of guaianes and agarofurans sesquiterpenes were higher in WQN compared with CQN. Ten guaianes and three agarofurans sesquiterpenes were observed in WQN, but only three guaianes and one agarofuran sesquiterpene were detected in CQN, as shown in Tables S2–S4. The results indicate that WQN possess not only a higher concentration, but also a greater chemical diversity of guaiane and agarfuran sesquiterpenes than CQN. To date, the chemical constituents of only two “Qi-Nan” agarwood varieties were reported. In 1991, a series of guaiane sesquiterpenes (**23**–**33**) featuring a 7-isopropenyl moiety, except for α-guaiene (**22**), were isolated and identified from kanankoh, a high-quality agarwood of *A. agallocha* [12]. Among them, compounds (**23**), (**25**–**28**), and (**33**) are oxidized at C-14, which is rarely encountered in nature. In this work, GC-MS identified guaiane sesquiterpenes (**23**), (**25**), and (**27**) in WQN, and (**26**) and (**27**) in CQN, indicating that guaiane sesquiterpenes with a 7-isopropenyl group are not uncommon in WQN and CQN agarwood. In 2016, six guaiane-furans (**35**–**40**) were isolated from a high-quality agarwood of *A. sinensis* named “Lv Qi-Nan” in Chinese [15]. These guaiane-furans sesquiterpenes possess a 5,11-epoxy ring with multiple stereoisomers, and are functionalized at C-15. None of these sesquiterpenes were found in the 22 samples of CQN agarwood but three of them (**35**, **37** and **38**) were detected in the WQN agarwood. This noteworthy difference indicated that guaiane-furan sesquiterpenes—despite the fact that two of which, sinenofuranol and sinenofuranal, were isolated from ordinary agarwood [28]—may be the identifying components of WQN allowing us to distinguish it from CQN. Both of the two aforementioned agarwood varieties were reported as “Qi-Nan” agarwood and were richer in guaiane sesquiterpenes compared with CQN, while the structural characteristics of guaiane sesquiterpenes in the two agarwood types were totally different, which may be due to their different origin plants. At present, **15** agarofuran sesquiterpenes have been reported from agarwood [23]. Only three (**10**, **11** and **12**) of them were isolated from the two above noted high-quality “Qi-Nan” agarwood varieties and others were identified from ordinary agarwood [23]. Although significant differences in the relative contents of agarofuran sesquiterpenes between WQN and CQN agarwood were observed in this study, it is hard to say that agarfuran sesquiterpenes could be a chemical marker for high quality agarwood. 

**Figure 4:**
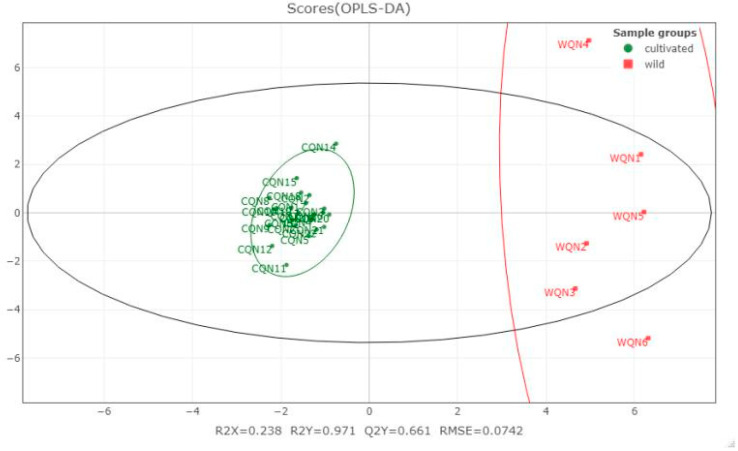
Orthogonal partial least-squares (OPLS) model of the chemical constituents distribution of cultivated and wild harvested “Qi-Nan” agarwood.

The compound information of CQN and WQN agarwood was introduced to the orthogonal partial least-squares (OPLS) statistical analysis, except for WQN7 which was formed in the bark, while the other samples were obtained in the xylem. The quality of the model was evaluated by R2X, R2Y and Q2Y. The R2X/R2Y value represents the percentage of predictor/response variance explained. The Q2Y refers to the predictive ability of the model which is calculated by cross-validation, and the RMSE represents the prediction accuracy of the model. As shown in Figure 4, The relatively low R2X value may be attributed to the scattered compound distribution of the WQN agarwood. Meanwhile, the compound distribution of CQN agarwood is centralized. This indicated that the quality uniformity of CQN agarwood is better than the WQN agarwood varieties, which were purchased from a wide range of geographical areas.

The R2Y, Q2Y and RMSE values proved that the prediction ability of the OPLS model is reliable in a certain degree, which means the WQN and CQN agarwood can be clearly distinguished. The variable importance in projection (VIP) value was then calculated and the VIP > one rule was applied. Table 1 lists the compounds with VIP values which may account for the difference in compound distribution between WQN and CQN agarwood. It can be seen that three 2-(2-phenylethyl)chromones were ranked in the five compounds that VIP > two which indicated these 2-(2-phenylethyl)chromones could be characteristic compounds, able to distinguish WQN agarwood from CQN agarwood.

### 2.2. The Fragrant Sesquiterpenes and 2-(2-Phenylethyl)chromones Identified in Agarwood

The scent is one of the most important factors to consider in determining the quality of agarwood. Generally, low quality agarwood possesses a low content of resin and high content of wood. When the low-quality agarwood is burning, it may release woody aromas which will irritate the eyes and nose. However, high-quality agarwood burns evenly and rather slowly while releasing its soft and pleasant scents gradually. Its fragrance lingers at the room for a longer period. Unlike ordinary agarwood, the scent of “Qi-Nan” agarwood can be smelled even at room temperature [29]. The specific odors of agarwood are closely correlated with their chemical compositions. We summarized the odor description of the constitutions in agarwood and have listed them in Table 2. Most of the odor components were sesquiterpenes. For instance, (−)-guaia-1(10),11-dien-15-al (**25**) has a pleasant β-damascenone-like woody and floral note with a slight cooling side note [10]. Many other sesquiterpenes (**1**, **4**, **5**, **18**, **19**, **34**, **35**, **36**, **37**, **38,** and **39**), in particular those only isolated from the above-mentioned two “Qi-Nan” agarwood, also have fresh floral, sweet, and cool scents, indicating that those sesquiterpenes were the main contributors to the gorgeous and elegant character of “Qi-Nan” agarwood. While the sesquiterpenes (**13**, **14**, **15**, **44**, **45**, **48**, **49**, etc.) reported only from the ordinary agarwood or from both the “Qi-Nan” agarwood and ordinary agarwood released a woody, or even strong woody scent. 2-(2-Phenylethyl)chromone derivatives were reported be responsible for the warm, sweet, balsamic, long lasting odor when agarwood is burnt or heated [5]. Of particular note here, is that two 2-(2-phenylethyl)chromones, (**59**) and (**60**) which account for the highest content in “Qi-Nan” agarwood, were odorless, but they yielded a pleasant scent when heated. They were subjected to pyrolysis into benzaldehyde and 4-methoxy benzaldehyde, respectively, in an air stream at 150 °C for 6 h [30] and these pyrolysis products, together with sesquiterpenes, composed the incense of agarwood [5]. The above information gives insight into the correlation between the scents and the compounds of agarwood, and provides the basis for the scents as an index to grade the quality of agarwood.

## 3. Materials and Methods

### 3.1. GC-MS Analysis

A Hewlett Packard GC 6890 gas chromatography instrument coupled with a Mass Selective Detector (5975C, Agilent Technologies, Wilmington, Delaware, USA) was used for the analysis. Separation of the samples by gas chromatography was carried out using a HP-5MS 5% Phenyl Methyl Siloxane column (30 m × 0.25 mm × 0.25 µm) (Phenomenex, Torrence, CA, USA). The parameters were set: injection volume, 1.0 µL; the front inlet temperature, 250 °C; split ratio, 40:1; the flow rate of the helium (carrier gas), 1.0 mL/min; the interface temperature, 280 °C; ionization of the compounds, electron impact (EI); emission current, 70 eV; the ion source temperature, 230 °C. The oven program commenced at 50 °C and increased to 310 °C at a rate of 5 °C/min, then held for 10 min. The spectra were obtained over the mass range of *m*/*z* 29 to 500 and the relative contents of the compounds were determined by normalization. The NIST14 database, WILEY275 database, and the mass spectroscopic data in the literature were used to characterize the compounds.

### 3.2. Plant Material

In the plantation, the “Qi-Nan” seedlings were obtained by the scions grafting method. The different kinds of excellent wild germplasm of *A. sinensis* discovered in Dianbai area were selected as parental plants, and the ordinary germplasm of *A. sinensis* was used as a grafting stock. The scions were usually one year to one and a half years old, 0.5–1.0 cm diameters branches of parental plants, with two or three buds on it. The upper branches of grafting stocks were cut off and only a 5–10 cm high main stem was left, then a 2–3 cm deep cut was made along the longitudinal direction of each main stem. Each scion was cut into a wedge shape and inserted into each rootstock cut, then tied up with a plastic film around the incision site. They were kept under 25 °C, 40–50% shading, and moisturizing conditions for about 30 days, then the graft seedlings began to germinate. When the graft seedlings were 2 or 3 years old, the agarwood formation was induced by holing the trunk. The twenty-two CQN agarwood samples were all harvested about ten months after holing the trunk. All of the 7 WQNs were obtained from two agarwood collectors. It is worth mentioning that, different from all the other samples obtained from the trunk of the plant, WQN7 was obtained from the bark of the trunk, which was named as “tabby cat striation” “Qi-Nan” for the appearance of its outside surface which looks like the skin of a tiger. All the agarwood samples produced a scent under room temperature. The odor descriptions and origin plants of these agarwood varieties were identified by Professor Dai Haofu and have been listed in Table 3. The samples are shown in Figures S1 and S2. The voucher specimens were deposited at the Institute of Tropical Bioscience and Biotechnology, Chinese Academy of Tropical Agricultural Sciences.

### 3.3. Sample Preparation

All the samples were ground into a powder and dried. The accurately weighed powder (2.00 g) was refluxed in ethanol (3 × 15 mL) and the ethanol extract was filtered and concentrated. The yields of the agarwood samples were listed in Table S1.

The accurately weighed agarwood powder (0.20 g) was extracted under ultrasonic wave (42 KHz, 20 min) with Et_2_O (3 × 30 mL) at 20 °C for three times. The Et_2_O extract was filtered and evaporated to obtain a brownish yellow oil. Then, the oil was dissolved in 2.0 mL methanol, filtered through 0.45 µm microporous filtering film and stored at 4 °C.

### 3.4. Orthogonal Partial Least-Squares (OPLS) Statistical Analysis

The compound information of CQN and WQN agarwood was scaled firstly, then calculated by the ropls R package and plotted by the plotly R package. For the reason of unique forming site of sample WQN7, the process did not contain it.

## 4. Conclusions

Recently, CQN has emerged as a new high-quality agarwood in the agarwood market, owing to its similar characteristics—such as high content of resin and richness in two 2-(2-phenylethyl)chromonederivatives, 2-(2-phenylethyl)chromone (**59**) and 2-[2-(4-methoxyphenyl)ethyl]chromone (**60**)—to the WQN variety. In this study, we compared the chemical constituents and fragrant components of two reported WQN agarwood samples from *A. agallocha* Roxb, and *A. sinensis*, respectively—with those of the CQN agarwood variety having been collected in Dianbai, Guangdong province, and ordinary agarwood, as well as the different analyzed samples of WQN agarwood and CQN agarwood having been collected in Dianbai, Guangdong province—by GC-MS, which revealed several noteworthy differences between the WQN and CQN agarwood varieties. The chemical diversity of WQN agarwood was greater than that of CQN agarwood. The content of (**59**) and (**60**) was higher in CQN agarwood than in WQN agarwood. For the sesquiterpenes, the richness and diversity of sesquiterpenes, particularly guaiane and agarofuran sesquiterpenes, in WQN agarwood was higher than in CQN agarwood. In addition, guaianefurans sesquiterpenes were only detected by GC-MS in WQN agarwood and could be a chemical marker for WQN agarwood. Moreover, we summarized the odor descriptions of the constituents, most of which were reported from WQN agarwood, and established the correlation of scents and chemical constituents in the agarwood, especially in the high-quality agarwood. In summary, the CQN agarwood possess similar characteristics, including high contents of alcohol extracts and two 2-(2-phenylethyl)chromone derivatives (**59** and **60**), which were the key criterions for the high quality agarwood, to the WQN agarwood. However, several noteworthy differences in chemical constituents and fragrant components were also found between the two agarwood varieties. These results are helpful for distinguishing the WQN agarwood from CQN agarwood collected in Dianbai, Guangdong province and the advancement of the agarwood industry.

## Figures and Tables

**Table 1:** Variable importance in projection (VIP) values calculated form OPLS model.

No.	Compound	Formula	VIP
1	5,8-Dihydroxy-2-(2-phenylethyl)chromone	C_17_H_14_O_4_	2.16
2	6-Hydroxy-2-(2-phenylethyl)chromone	C_17_H_14_O_3_	2.16
3	6,7-Dimethoxy-2-(2-phenylethyl)chromone	C_19_H_18_O_4_	2.16
4	2,t-3-Dimethyl-r-2-(3-methyl-2-butenyl)-1-cyclohexanone	C_13_H_22_O	2.12
5	(1β,4aβ,7β,8aβ)-Octahydro-7-[1-(hydroxymethyl)ethenyl]-1,8a-dimethylnaphthalen-4a(2*H*)-ol	C_15_H_26_O_2_	2.05
6	(+)-9-Hydroxy-selina-4,11-dien-14-al	C_15_H_22_O_2_	1.94
7	Kusunol (Valerianol)	C_15_H_26_O	1.77
8	Eremophila-9,11(13)-dien-12-ol	C_15_H_24_O	1.59
9	(*R*)-3,7-Dimethyl-6-Octenal	C_10_H_18_O	1.57
10	Citronellal	C_10_H_18_O	1.57
11	*D*-Limonene	C_10_H_16_	1.57
12	(−)-9-Hydroxy-selina-3,11-dien-14-al	C_15_H_22_O_2_	1.47
13	Neopetasane	C_15_H_22_O	1.44
14	8-Hydroxy-2-(2-phenylethyl)chromone	C_17_H_14_O_3_	1.41
15	Valerenol	C_15_H_24_O	1.24
16	11-Hydroxy-valenc-1(l0)-en-2-one	C_15_H_24_O_2_	1.17

**Table 2:** The odor sesquiterpenes and 2-(2-phenylethyl)chromones identified in agarwood.

No.	Compound	Odor Description	“Qi-Nan” from *A. agallocha*	“Qi-Nan” from *A. sinensis*	Ordinary Agarwood	Ref.
1	(5*S*,7*S*,10*S*)-(−)-Selina-3,11-dien-9-one	Fresh, sweet, reminiscent of blooming flowers	√			[10]
2	(5*S*,7*S*,9S,10*S*)-(+)-Selina-3,11-dien-9-ol	Woody, sweet, rather weak	√			[10]
3	Selina-3,11-dien-14-ol	Floral, herbaceous, minty			√	[31]
4	Selina-3,11-dien-14-al	Floral, woody, nuance of smoky sandalwood	√			[11]
5	Selina-4,11-dien-14-al	Pennyroyal-like, minty	√			[11]
6	10-epi-γ-Eudesmol	Camphor-like, woody, pelargonium			√	[32]
10	4,15-Dihydroxydihydro-β-agarofuran	balsamic note with a slightly cooling, like the smell of camphor		√		[14]
13	(+)-(4*S*,5*R*)-Dihydrokaranone	Strong woody, slightly camphoraceous, fumigating note, remarkable, intense, woody	√		√	[33], [34]
14	(+)-(4*S*,5*R*)-Karanone	Woody, amber-like, elegant	√		√	[33], [35]
15	Eremophila-9,11-dien-8-one(Neopetasane)	Sweet, woody, like dihydrokaranone, important oriental, fumigating character		√	√	[11]
16	8,12-Epoxyeremophila- 9,11(13)-diene	Pleasant woody with vetiver character			√	[36]
18	7β-*H*-9(10)-ene-11,12-epoxy-8-oxoeremophilane	Fresh floral, fumigating		√		[14]
19	7α-*H*-9(10)-ene-11,12-epoxy-8-oxoeremophilane	Powerful long-lasting pennyroyal-like minty smell		√		[14]
20	Dehydro-jinkoh-eremol	Woody, slightly balsamic, bitter	√			[11]
23	(−)-Guaia-1(10),11-dien-15,2-olide	Powerful, long lasting, woody, sweet note	√			[13]
25	(−)-Guaia-1(10),11-dien-15-al	Pleasant, β-damascenone-like, woody, touch of camphor	√			[10]
27	(−)-Guaia-1(10),11-diene-15-carboxylic acid	Weak woody, harmonizes other compounds by heating	√			[31]
34	Qinanol A	Cool minty, sweet note		√		[15]
35	Qinanol B	Floral, elegant		√		[15]
36	Qinanol C	Slightly spicy, cool minty, rather weak		√		[15]
37	Qinanol D	Fresh floral, refreshing cool, slightly sweet note		√		[15]
38	Qinanol E	Long lasting refreshing cool, strong balsamic		√		[15]
39	Sinenofuranol	Strong camphoraceous, sweet note		√	√	[15]
40	Guaiol	Rosy, powdery			√	[32]
44	Agarospirol	Spicy, peppery, woody	√		√	[5]
45	Baimuxinal	Woody, balsamic	√		√	[33]
46	Vetispira-2(11),6-dien-14-al	Woody, sweet, smoky, phenolic, typical agarwood, quite weak			√	[36]
47	2,14-Epoxy-vetispir-6-ene	Nearly odourless			√	[5]
48	Jinkohol	Extremely strong woody			√	[37]
49	Jinkohol II	Woody, slightly campheraceous, typical agarwood, when heated			√	[38]
50	4-epi-10-Hydroxyacoronene	Extremely strong spicy, slightly smoky		√		[14]
51	4-epi-15-Hydroxyacorenone	Fir flavor, slightly refreshing cool, bitter, smoky		√		[14]
52	Hydroxycalamenene	Extremely strong sweet		√		[39]
59	2-(2-Phenylethyl)chromone	Lasting pleasant odor of agarwood when burned as incenses	√	√	√	[30]
60	2-(2-(4-methoxyphenyI)ethyl)chromone	Lasting pleasant odor of agarwood when burned as incenses	√	√	√	[30]

**Table 3:** The sample information.

No.	Odor Descriptions	Species	Place of Production	Induction Method	Cultivated or Wild
CQN1	Woody, slightly spicy	*Aquilaria sinensis*	Dianbai, Guangdong province	Holing	cultivated
CQN2	Strong woody, sweet cooling note				
CQN3	Weak woody and cooling note				
CQN4	Weak woody and sweet note				
CQN5	Weak woody and cooling note				
CQN6	Slightly woody				
CQN7	Slightly woody and grease smell,				
CQN8	Strong woody, fresh floral, fumigating note, sweet and cooling smell				
CQN9	Slightly woody				
CQN10	Weak floral note				
CQN11	Woody with sweet note				
CQN12	Powerful woody, slightly spicy				
CQN13	Woody, sweet note				
CQN14	Weak sweet and floral smell				
CQN15	Powerful, long lasting, woody and cooling note				
CQN16	Slightly woody and sweet note				
CQN17	Woody with slightly cooling minty				
CQN18	Strong floral and sweet note				
CQN19	Woody				
CQN20	Remarkable floral, sweet note with slightly woody				
CQN21	Woody, slightly sweet note				
CQN22	Weak sweet and woody				
WQN1	Strong woody, cooling, sweet note	*Aquilaria* spp.	Vietnam	nature	wild
WQN2	Strong woody, sweet note	*Aquilaria* spp.	Vietnam	nature	wild
WQN3	Strong woody, cooling, sweet note	*Aquilaria sinensis*	Hainan province	nature	wild
WQN4	Powerful floral smell, slightly woody	*Aquilaria sinensis*	Hainan province	nature	wild
WQN5	Strong cooling, sweet note with slightly spicy	*Aquilaria* spp.	Vietnam	nature	wild
WQN6	Strong cooling, sweet note	*Aquilaria sinensis*	Hong Kong	nature	wild
WQN7	Weak woody, sweet note	*Aquilaria sinensis*	Hong Kong	nature	wild

## Data Availability

The data presented in this study are available in Supplementary Material.

## Supplementary Materials

The following are available online; the data contain the samples of cultivated (CQN12-CQN22, Figure S1) and wild harvested (WQN1-WQN7, Figure S2) “Qi-Nan” agarwood. The yields of the agarwood samples were listed in Table S1. The constituents and their structures identified in cultivated (Tables S2, S3 and Figure S3) and wild harvested (Table S4 and Figure S4) “Qi-Nan” agarwood by GC-MS.
