# Supplementary material for: The Characteristic Fragrant Sesquiterpenes and 2-(2-Phenylethyl)chromones in Wild and Cultivated “Qi-Nan” Agarwood
Source: Molecules. 2021 Jan 15;26(2):436. doi: 10.3390/molecules26020436 (PMC7830084; doi:10.3390/molecules26020436)
Supplement: Supplementary file 1 [file molecules-26-00436-s001.pdf]

Supporting information for

The characteristic fragrant sesquiterpenes and 2-(2-phenylethyl)chromones in wild and cultivated "Qi-Nan" agarwood

Li Yang [1,2,3,†], Jin-ling Yang [1,2,3, †], Wen-Hua Dong [1,2,3], Ya-Li Wang [1,2,3], Jun Zeng [1,2,3], Hao Wang [1,2,3], Wen-Li Mei [1,2,3*] and Hao-Fu Dai [1,2,3*]

[1] Hainan Engineering Research Center of Agarwood, Institute of Tropical Bioscience and Biotechnology, Chinese Academy of Tropical Agricultural Sciences, Haikou 571101, China; yangli@itbb.org.cn (L.Y.); jinlyang@126.com (J.-L.Y.); dongwenhua@itbb.org.cn (W.-H.D.); wyl200881@163.com (Y.L.W.); zengjun@itbb.org.cn (J.Z.); wanghao@itbb.org.cn (H.W.)

[2] Hainan Key Laboratory for Research and Development of Natural Products from Li folk Medicine, Institute of Bioscience and Biotechnology, Chinese Academy of Agricultural Sciences, Haikou 571101, China

[3] Hainan Institute for Tropical Agricultural Resources, Chinese Academy of Agricultural Sciences, Haikou 571101, China

* Correspondence: meiwenli@itbb.org.cn (W.-L.M.); daihaofu@itbb.org.cn (H.-F.D.)

† These authors contributed equally to this work.

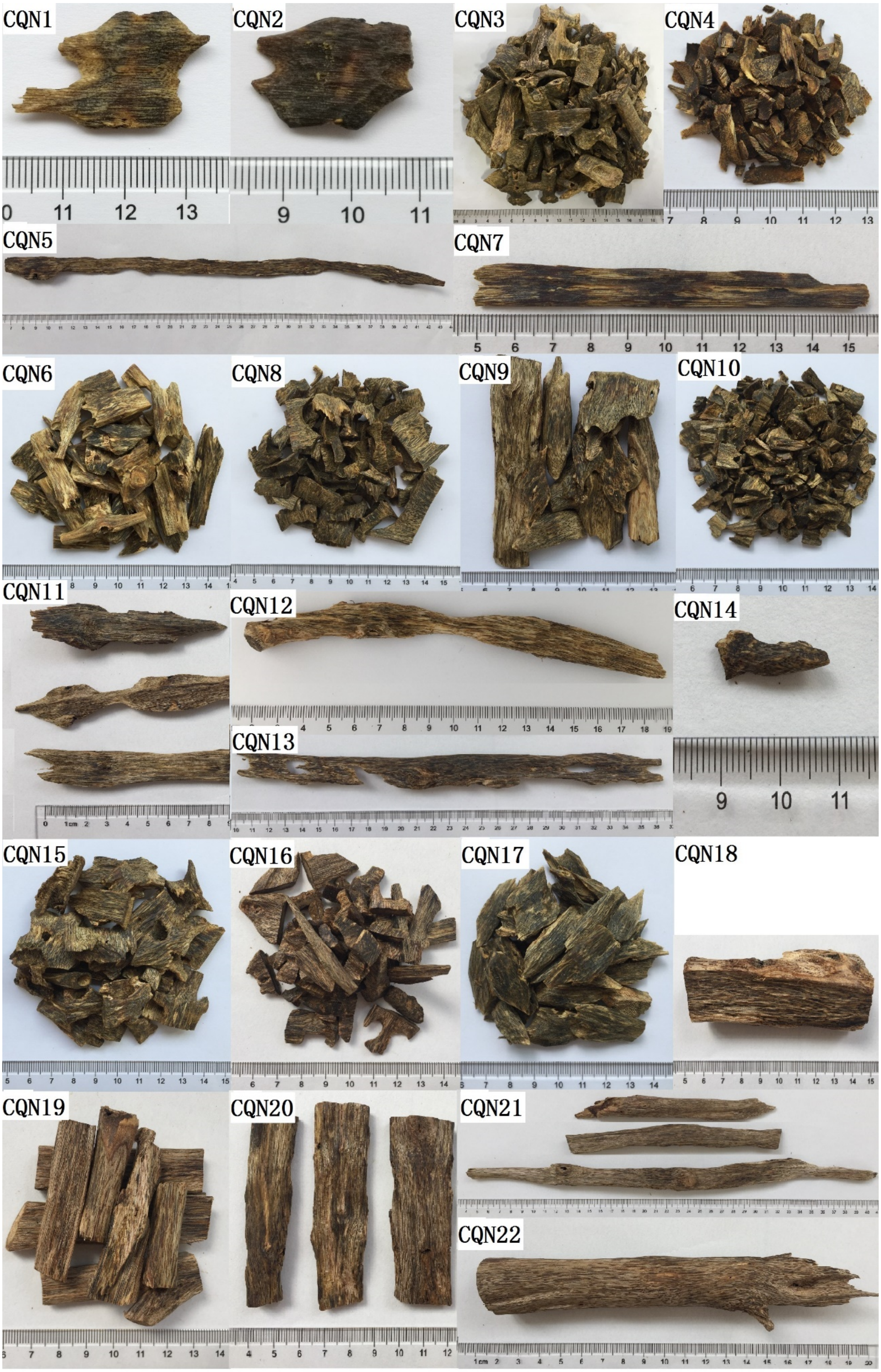

CQN11

Figure 1. The cultivated Qi-Nan agarwood.

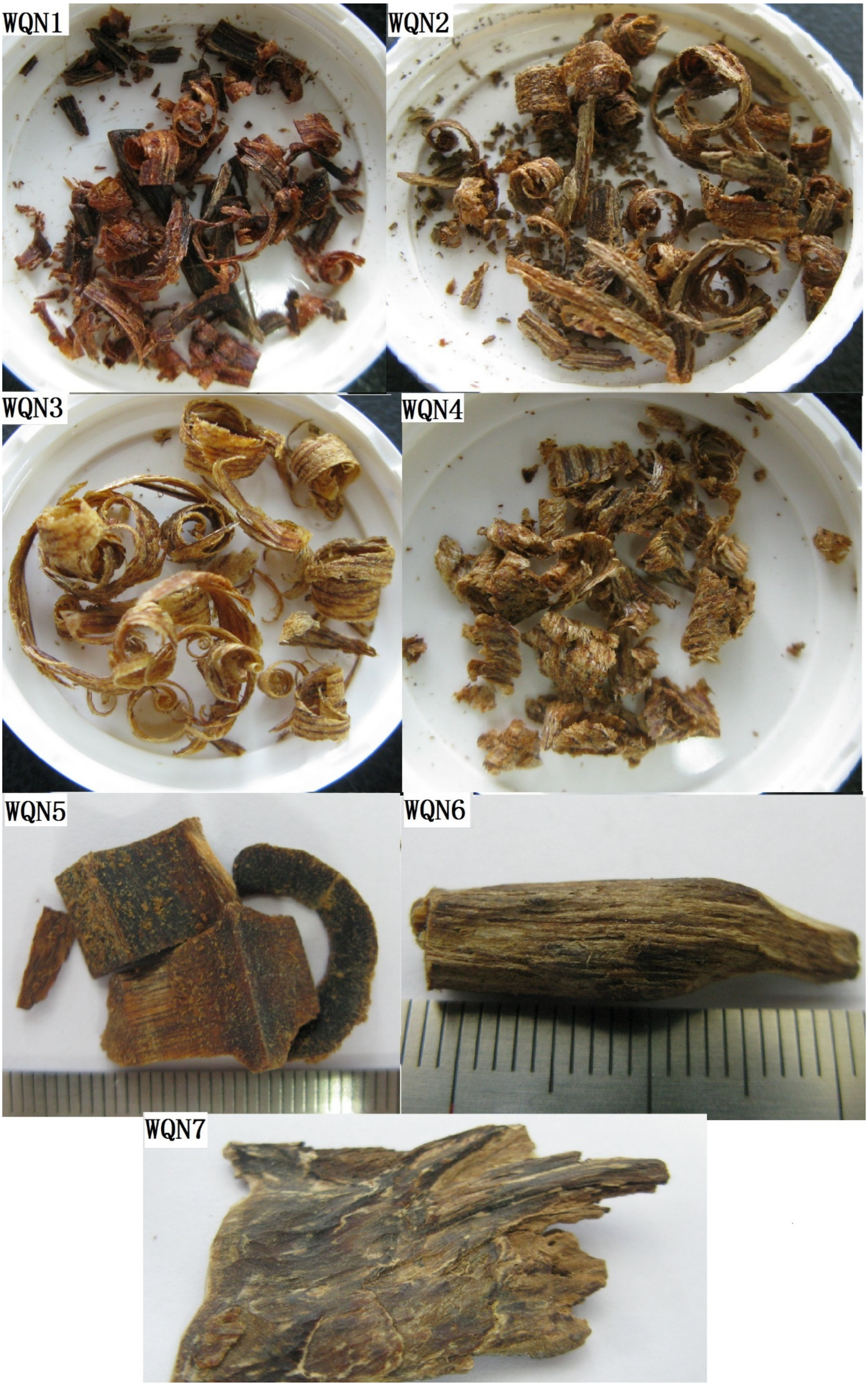

Figure 2. The wild harvested Qi-Nan agarwood.

Table 1. Ethanol extract contents of 22 CQN agarwood and 7 WQN agarwood.

**CQN1**	**CQN2**	**CQN3**	**CQN4**	**CQN5**	**CQN6**	**CQN7**	**CQN8**	**CQN9**	**CQN10**
64.73	66.71	49.60	57.39	47.61	56.62	47.73	52.57	35.78	48.05
CQN11	CQN12	CQN13	CQN14	CQN15	CQN16	CQN17	CQN18	CQN19	CQN20
50.01	41.03	47.69	60.25	47.73	45.24	45.88	36.75	28.68	44.19
CQN21	CQN22	WQN1	WQN2	WQN3	WQN4	WQN5	WQN6	WQN7	
29.11	20.09	-	-	36.40	60.13	-	-	53.01	

not determined due to the limited amount of samples.

Table 2. Chemical constituents detected in cultivated Qi-Nan agarwood (CQN1-CQN11) by GC-MS.

**No.**	**RT**	**Compound**	**Formula**	***m/z***	**CQN1**	**CQN2**	**CQN3**	**CQN4**	**CQN5**	**CQN6**	**CQN7**	**CQN8**	**CQN9**	**CQN10**	**CQN11**
1	12.04	Benzylacetone	$C_{10}H_{12}O$	148		0.38									
2	12.29	4-Methoxy-benzaldehyde	$C_8H_8O_2$	136										0.49	
3	18.83	2,4-di-*tert*-Butylphenol	$C_{14}H_{22}O$	206											
4	18.97	2-Methylchromone	$C_{10}H_8O_2$	160								0.45	0.50	0.97	
5	20.40	α-Santalol	$C_{15}H_{24}O$	220	0.30								0.95		1.30
6	21.92	(−)-Guaia-1(10),11-dien-15-ol□	$C_{15}H_{24}O$	220											0.42
7	21.96	β-Eudesmol◇	$C_{15}H_{26}O$	222											
8	22.04	α-Eudesmol◇	$C_{15}H_{26}O$	222											
9	22.06	Selin-6-en-4α-ol◇	$C_{15}H_{26}O$	222											
10	23.43	(*S*)-4a-Methyl-2-(1-methylethyl)-3,4,4a,5,6,7-hexahydronaphthalene◇	$C_{14}H_{22}$	190										0.94	
11	24.02	Kusunol (Valerianol)■	$C_{15}H_{26}O$	222								0.50			
12	24.16	Neopetasane■	$C_{15}H_{22}O$	218	0.74	0.64	0.66	0.85	0.46	0.64	0.53	1.20	0.55	0.88	
13	24.27	(1β,4aβ,7β,8aβ)-Octahydro-7-[1-(hydroxymethyl)ethenyl]-1,8a-dimethylnaphthalen-4a(2H)-ol■	$C_{15}H_{26}O_2$	238	0.28	0.37									
14	24.58	(-)-7βH-Eudesmane-4α,11-diol◇	$C_{15}H_{28}O_2$	240											
15	25.25	Dihydrokaranone■	$C_{15}H_{22}O$	218											
16	25.36	Valenca-1(10),8-dien-11-ol■	$C_{15}H_{24}O$	220	0.29	0.54		0.58	0.54			0.57	0.56	1.60	1.42
17	25.74	2,t-3-Dimethyl-r-2-(3-methyl-2-butenyl)-1-cyclohexanone■	$C_{13}H_{22}O$	194	1.10	1.08	1.06	1.26	0.80	0.96	1.02	1.42	0.68	1.13	0.54
18	25.88	Baimuxinal☆	$C_{15}H_{24}O_2$	236	0.38	0.58	0.65	2.12	2.80	1.61	2.18	2.26	1.21	0.65	1.17
19	26.26	Isobaimuxinol★	$C_{15}H_{26}O_2$	238										0.47	

20	26.65	Selina-4,11-dien-14-oic acid◇	$C_{15}H_{22}O_2$	234				0.86	0.77	1.08	0.86	1.83	1.40	1.37	2.45
21	26.96	(−)-2*α*-Hydroxyguaia-1(10),11-dien-15-oic acid□	$C_{15}H_{22}O_3$	250	0.15	0.10	0.18								
22	26.99	2-((2R,4aR,8aS)-4a-Methyl-8-methylenedecahydronaphthalen-2-yl)prop-2-en-1-ol◇	$C_{15}H_{24}O$	220											
23	27.63	[4aR-(4a*α*,6*α*,8aβ)]-4a,5,6,7,8,8a-Hexahydro-6-[1-(hydroxymethyl)ethenyl]-4,8a-dimethyl-2(1H)-naphthalenone◇	$C_{15}H_{22}O_2$	234								0.49			0.90
24	27.77	(3a*R*,4a*S*,5*R*,9a*S*)-5,8-Dimethyl-3-methylene-3a,4,4a,5,6,7,9,9a-octahydroazuleno[6,5-b]furan-2(3H)-one □	$C_{15}H_{20}O_2$	232											
25	28.26	Karanone■	$C_{15}H_{20}O$	216	1.95	1.98	0.68	2.16	2.01	0.79	1.28	1.48	1.59		1.55
26	28.27	12,15-Dioxo-α-selinen◇	$C_{15}H_{20}O_2$	232										1.29	0.44
27	28.48	7*α*-*H*-9(10)-ene-11,12-Epoxy-8-oxoeremophilane■	$C_{15}H_{22}O_2$	234			0.21	0.80	1.09	0.66	0.81	1.05	0.83	1.86	
28	28.73	11-Hydroxy-valenc-1(l0)-en-2-one■	$C_{15}H_{24}O_2$	236				0.59	0.73						
29	28.75	(−)-9-Hydroxy-selina-3,11-dien-14-al◇	$C_{15}H_{22}O_2$	234	1.18	1.38						0.62			0.46
30	28.89	Eudesma-5,11(13)-dien-8,12-olide◇	$C_{15}H_{20}O_2$	232	0.29				1.25		0.76	1.20	1.32		1.24
31	29.97	(+)-9-Hydroxy-selina-4,11-dien-14-al◇	$C_{15}H_{22}O_2$	234											
32	31.65	9a-Octahydro-4-hydroxy-4a,5-dimethyl-3-methylene-3a,4,4a,5,6,7,9,3H-naphtho[2,3-b]furan-2-one■	$C_{15}H_{20}O_3$	248	0.31			0.53	0.72	0.56	0.41	1.03	0.77	0.73	1.80
33	33.14	2-[2-(2-Hydroxyphenyl)ethyl]chromone	$C_{17}H_{14}O_3$	266	0.70	0.75					0.44				
34	35.06	2-(2-Phenylethyl)chromone	$C_{17}H_{14}O_2$	250	23.42	21.93	33.49	28.46	32.81	23.49	27.88	28.06	37.35	30.89	37.85
35	38.62	2-[2-(4-Methoxyphenyl)ethyl]chromone	$C_{18}H_{16}O_3$	280	54.11	50.50	56.43	51.52	46.47	53.44	50.57	44.88	44.64	46.58	39.90
36	39.51	2-[2-(3-Hydroxyphenyl)ethyl]chromone	$C_{17}H_{14}O_3$	266	0.50			0.05	0.60		0.35		0.25		0.54
37	40.29	2-[2-(4-Hydroxyphenyl)ethyl]chromone	$C_{17}H_{14}O_3$	266	0.51	0.63		0.64	0.67	0.66	0.64	0.47	0.49	0.46	0.40
38	41.24	2-[2-(3-Methoxy-4-hydroxyphenyl)ethyl] chromone	$C_{18}H_{16}O_4$	296	3.15	3.85	5.28	1.32	1.29	2.68	2.08	0.80	1.39	1.03	1.03
39	41.53	New chromone※	$C_{18}H_{18}O_4$	310	0.56			0.10	0.35	0.48	0.51		0.35	0.05	0.43
40	41.96	2-[2-(3-Hydroxy-4-methoxyphenyl)ethyl] chromone	$C_{18}H_{16}O_4$	296	9.43	11.56	0.96	7.30	3.95	12.49	9.23	7.51	5.14	5.86	5.18
		■：the relative content of eremophilane sesquiterpenes			4.67	4.61	2.61	6.77	6.35	3.61	4.05	7.25	4.98	6.20	5.31
		The number of eremophilane sesquiterpenes			6	5	4	7	7	5	5	7	6	5	4
		□：the relative content of guaiane sesquiterpenes			0.15	0.10	0.18	0	0	0	0	0	0	0	0.42
		The number of guaiane sesquiterpenes			1	1	1	0	0	0	0	0	0	0	1
		◇：the relative content of eudesmane sesquiterpenes			1.47	1.38	0	0.86	2.02	1.08	1.62	4.14	2.72	3.60	5.49

The number of eudesmane sesquiterpenes	2	1	0	1	2	1	2	4	2	3	5
★: The relative content of agarofurans sesquiterpenes	0	0	0	0	0	0	0	0	0	0.47	0
The number of agarofurans sesquiterpenes	0	0	0	0	0	0	0	0	0	1	0
☆: the relative content of agarospirane sesquiterpenes	0.38	0.58	0.65	2.12	2.80	1.61	2.18	2.26	1.21	0.65	1.17
The number of agarospirane sesquiterpenes	1	1	1	1	1	1	1	1	1	1	1
the relative content of all sesquiterpenes	6.97	6.67	3.44	9.75	11.17	6.30	7.85	13.65	9.86	10.92	13.69
The number of all sesquiterpenes	11	8	6	9	10	7	8	12	10	10	12
the relative content of chromones	92.38	89.22	96.16	89.39	86.14	93.24	91.70	81.72	89.61	84.87	85.33
the number of chromones	8	6	4	7	7	6	8	5	7	6	7
the relative content of No.34 and No.35	77.53	72.43	89.92	79.98	79.28	76.93	78.45	72.94	81.99	77.47	77.75
the relative content of other chromones	14.85	16.79	6.24	9.41	6.86	16.31	13.25	8.78	7.62	7.40	7.58
the number of other chromones	6	4	2	5	5	4	6	3	5	4	5
Total	99.35	96.27	99.60	99.14	97.31	99.54	99.55	95.82	99.97	97.25	99.02

Note: ◇: eudesmane sesquiterpene; □: guaiane sesquiterpene;☆: agarospirane sesquiterpene ; ■: eremophilane sesquiterpene;★: agarofurans sesquiterpenes.※:a new chromone which two methoxy were observed on benzyl moiety.

Table 3. Chemical constituents detected in cultivated Qi-Nan agarwood (CQN12-CQN22) by GC-MS.

No.	RT	Compound	Formula	*m/z*	CQN12	CQN13	CQN14	CQN15	CQN16	CQN17	CQN18	CQN19	CQN20	CQN21	CQN22
1	12.04	Benzylacetone	$C_{10}H_{12}O$	148					0.50	0.61		0.62			
2	12.29	4-Methoxy-benzaldehyde	$C_8H_8O_2$	136											
3	18.83	2,4-di-*tert*-Butylphenol	$C_{14}H_{22}O$	206			0.52								
4	18.97	2-Methylchromone	$C_{10}H_8O_2$	160											
5	20.40	α-Santalol	$C_{15}H_{24}O$	220	1.89				0.68	0.51		1.15	1.53	1.28	0.74
6	21.92	(−)-Guaia-1(10),11-dien-15-ol□	$C_{15}H_{24}O$	220											
7	21.96	β-Eudesmol◇	$C_{15}H_{26}O$	222											0.67
8	22.04	α-Eudesmol◇	$C_{15}H_{26}O$	222											0.90
9	22.06	Selin-6-en-4α-ol◇	$C_{15}H_{26}O$	222								0.66			
10	23.43	(*S*)-4a-Methyl-2-(1-methylethyl)-3,4,4a,5,6,7-hexahydronaphthalene◇	$C_{14}H_{22}$	190				1.03		1.11		0.55			
11	24.02	Kusunol (Valerianol)■	$C_{15}H_{26}O$	222			0.86	0.61							
12	24.16	Neopetasane■	$C_{15}H_{22}O$	218		0.53	1.46	0.88	0.84		0.90	0.60			

13	24.27	(1β,4aβ,7β,8aβ)-Octahydro-7-[1-(hydroxymethyl)ethenyl]-1,8a-dimethylnaphthalen-4a(2H)-ol■	$C_{15}H_{26}O_2$	238			0.61								
14	24.58	(−)-7βH-Eudesmane-4α,11-diol◇	$C_{15}H_{28}O_2$	240											0.67
15	25.25	Dihydrokaranone■	$C_{15}H_{22}O$	218			0.80								
16	25.36	Valenca-1(10),8-dien-11-ol■	$C_{15}H_{24}O$	220	0.42		1.30	0.71	0.58	0.63		0.64	0.71	0.63	1.03
17	25.74	2,t-3-Dimethyl-r-2-(3-methyl-2-butenyl)-1-cyclohexanone■	$C_{13}H_{22}O$	194	0.50	0.71	2.59	1.04	1.06		1.42	0.88	0.69	0.67	
18	25.88	Baimuxinal☆	$C_{15}H_{24}O_2$	236	0.60	0.55	3.65	1.92	1.74	1.13	1.93	0.74			
19	26.26	Isobaimuxinol★	$C_{15}H_{26}O_2$	238			1.00								
20	26.65	Selina-4,11-dien-14-oic acid◇	$C_{15}H_{22}O_2$	234	4.00	0.79	0.93	0.79	1.66	0.95		3.29		2.83	0.95
21	26.96	(−)-2*α*-Hydroxyguaia-1(10),11-dien-15-oic acid□	$C_{15}H_{22}O_3$	250			0.42		0.69			0.60		1.30	
22	26.99	2-((2R,4aR,8aS)-4a-Methyl-8-methylenedecahydronaphthalen-2-yl)prop-2-en-1-ol◇	$C_{15}H_{24}O$	220							1.15		0.60		1.29
23	27.63	[4aR-(4a*α*,6*α*,8aβ)]-4a,5,6,7,8,8a-Hexahydro-6-[1-(hydroxymethyl)eThenyl]-4,8a-dimethyl-2(1H)-Naphthalenone◇	$C_{15}H_{22}O_2$	234	0.87									0.67	
24	27.77	(3a*R*,4a*S*,5*R*,9a*S*)-5,8-Dimethyl-3-methylene-3a,4,4a,5,6,7,9,9a-octahydroazuleno[6,5-b]furan-2(3H)-one□	$C_{15}H_{20}O_2$	232										0.63	
25	28.26	Karanone■	$C_{15}H_{20}O$	216	4.46	0.57	0.53	1.81	0.91		0.95	0.89		0.66	1.21
26	28.27	12,15-Dioxo-α-selinen◇	$C_{15}H_{20}O_2$	232	0.42					0.56				1.05	
27	28.48	7*α*-*H*-9(10)-ene-11,12-Epoxy-8-oxoeremophilane■	$C_{15}H_{22}O_2$	234			0.77	1.84	0.95	1.28	0.29				
28	28.73	11-Hydroxy-valenc-1(l0)-en-2-one■	$C_{15}H_{24}O_2$	236			0.43		0.50						
29	28.75	(−)-9-Hydroxy-selina-3,11-dien-14-al◇	$C_{15}H_{22}O_2$	234											
30	28.89	Eudesma-5,11(13)-dien-8,12-olide◇	$C_{15}H_{20}O_2$	232					0.75		0.84	1.26			1.53
31	29.97	(+)-9-Hydroxy-selina-4,11-dien-14-al◇	$C_{15}H_{22}O_2$	234											

32	31.65	9a-Octahydro-4-hydroxy-4a,5-dimethyl-3-methylene-3a,4,4a,5,6,7,9,3H-naphtho[2,3-b]furan-2-one■	$C_{15}H_{20}O_3$	248	1.14		0.42		0.87						
33	33.14	2-[2-(2-Hydroxyphenyl)ethyl]chromone	$C_{17}H_{14}O_3$	266	0.50				0.50			0.56			
34	35.06	2-(2-Phenylethyl)chromone	$C_{17}H_{14}O_2$	250	39.11	41.50	40.47	45.50	46.97	50.49	57.29	54.63	59.19	58.55	66.45
35	38.62	2-[2-(4-Methoxyphenyl)ethyl]chromone	$C_{18}H_{16}O_3$	280	39.24	50.29	40.85	41.39	38.50	39.47	31.24	31.61	36.42	29.07	20.45
36	39.51	2-[2-(3-Hydroxyphenyl)ethyl]chromone	$C_{17}H_{14}O_3$	266											
37	40.29	2-[2-(4-Hydroxyphenyl)ethyl]chromone	$C_{17}H_{14}O_3$	266	0.50	0.51									
38	41.24	2-[2-(3-Methoxy-4-hydroxyphenyl)ethyl] chromone	$C_{18}H_{16}O_4$	296	0.77	0.35						0.56			
39	41.53	New chromone※	$C_{18}H_{18}O_4$	310											
40	41.96	2-[2-(3-Hydroxy-4-methoxyphenyl)ethyl] chromone	$C_{18}H_{16}O_4$	296	4.23	3.55	0.69	0.79	0.57						
		■: The relative content of eremophilane sesquiterpenes			6.52	1.81	9.77	6.89	5.71	1.91	3.56	3.01	1.40	1.96	2.24
		The number of eremophilane sesquiterpenes			4	3	10	6	7	2	4	4	2	3	2
		□: The relative content of guaiane sesquiterpenes			0	0	0.42	0	0.69	0	0	0.60	0	1.93	0
		The number of guaiane sesquiterpenes			0	0	1	0	1	0	0	1	0	2	0
		◇: The relative content of eudesmane sesquiterpenes			5.29	0.79	0.93	1.82	2.41	2.62	1.99	5.76	0.60	4.55	6.01
		The number of eudesmane sesquiterpenes			3	1	1	2	2	3	2	4	1	3	6
		★: The relative content of agarofurans sesquiterpenes			0	0	1.00	0	0	0	0	0	0	0	0
		The number of agarofurans sesquiterpenes			0	0	1	0	0	0	0	0	0	0	0
		☆: The relative content of agarospirane sesquiterpenes			0.60	0.55	3.65	1.92	1.74	1.13	1.93	0.74	0	0	0
		The number of agarospirane sesquiterpenes			1	1	1	1	1	1	1	1	0	0	0
		The relative content of all sesquiterpenes			14.30	3.15	15.77	10.63	11.23	6.17	7.48	11.26	3.53	9.72	8.99

The number of all sesquiterpenes	9	5	14	9	12	7	7	11	4	9	9
The relative content of chromones	84.35	96.20	82.01	87.68	86.54	89.96	88.53	87.36	95.61	87.62	86.90
The number of chromones	6	5	3	3	4	2	2	4	2	2	2
The relative content of No.34 and No.35	78.35	91.79	81.32	86.89	85.47	89.96	88.53	86.24	95.61	87.62	86.90
The relative content of other chromones	6.00	4.41	0.69	0.79	1.07	0	0	1.12	0	0	0
The number of other chromones	4	3	1	1	2	0	0	2	0	0	0
Total	98.65	99.35	98.30	98.31	98.27	96.74	96.01	99.24	99.14	97.34	95.89

Note: ◇: eudesmane sesquiterpene; □: guaiane sesquiterpene;☆: agarospirane sesquiterpene ; ■: eremophilane sesquiterpene;★: agarofurans sesquiterpenes.※:a new chromone which two methoxy were observed on benzyl moiety

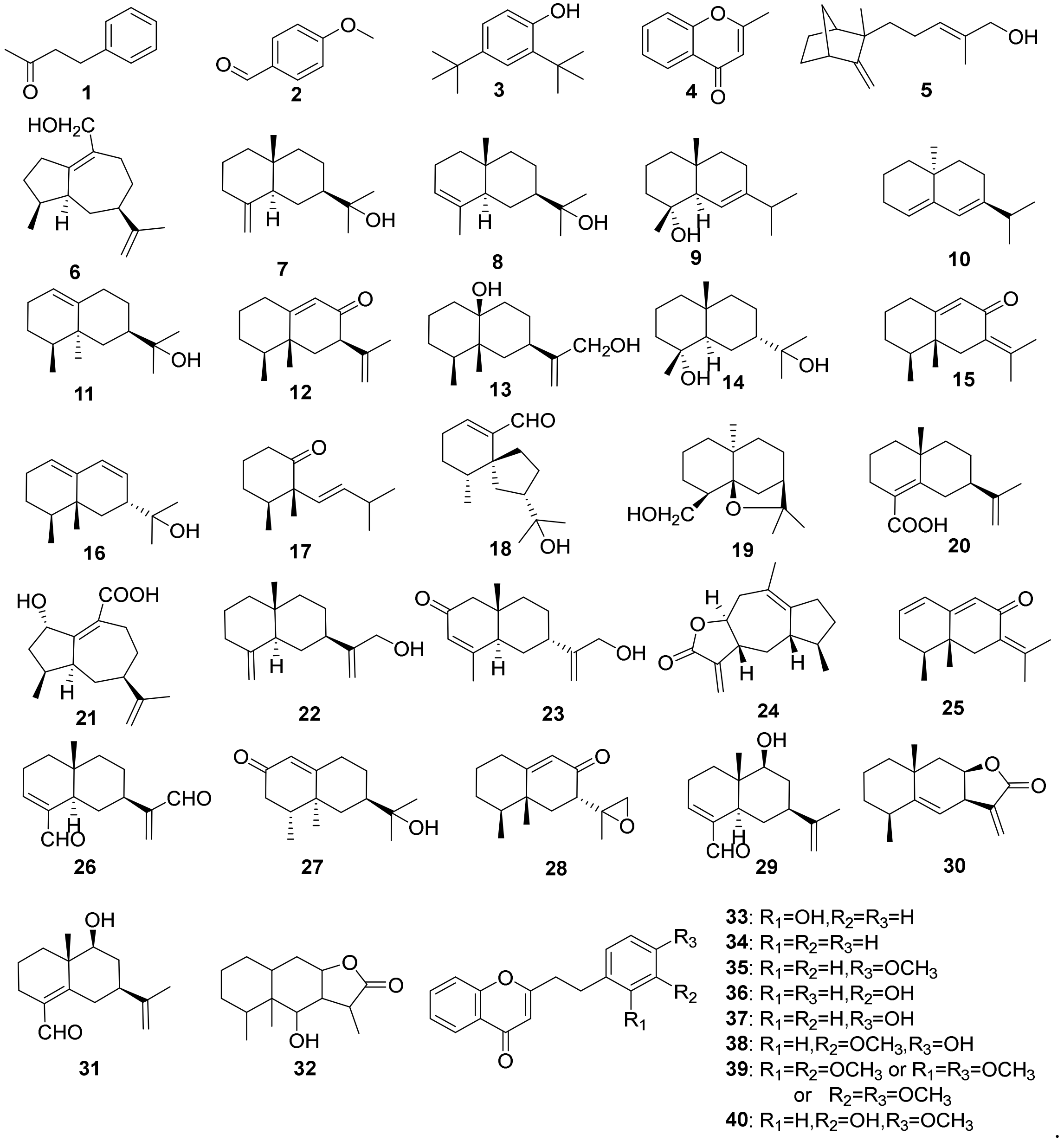

Figure 4. Structures of compounds identified in cultivated Qi-Nan agarwood (CQN12-CQN22) by GC-MS.

Table 4. Chemical constituents detected in wild harveated Qi-Nan agarwood by GC-MS.

No.	RT	Compound	Formula	*m/z*	WQN1	WQN2	WQN3	WQN4	WQN5	WQN6	WQN7
1	7.99	D-Limonene	$C_{10}H_{16}$	136						4.18	1.06
2	11.05	Citronellal	$C_{10}H_{18}O$	154						3.00	0.90
3	11.58	(R)3,7-Dimethyl-6-Octenal	$C_{10}H_{18}O$	154						1.65	0.67
4	21.37	Globulol□	$C_{15}H_{26}O$	222							1.45
5	22.23	Dihydro-*β*-agarofuran★	$C_{15}H_{26}O$	222							0.44
6	23.12	3-Methyl-4-(1,3,3-trimethyl-7-oxabicyclo [4.1.0]heptan-1-yl)-3-buten-2-one	$C_{14}H_{22}O_2$	222							0.50
7	23.43	(*S*)-4a-Methyl-2-(1-methylethyl)-3,4,4a,5,6,7-hexahydronaphthalene◇	$C_{14}H_{22}$	190					0.93		
8	23.87	Agarospirol☆	$C_{15}H_{26}O$	222							2.01
9	24.02	Kusunol (Valerianol) ■	$C_{15}H_{26}O$	222		0.49		0.77	0.62		
10	24.06	(-)-Aristolene■	$C_{15}H_{24}$	204							0.85
11	24.16	Neopetasane■	$C_{15}H_{22}O$	218			0.81	0.99			
12	24.23	Eremophila-9,11(13)-dien-12-ol■	$C_{15}H_{24}O$	220		0.54		1.10	0.71		
13	24.24	Guaiol□	$C_{15}H_{26}O$	222							1.34
14	24.27	(1β,4aβ,7β,8aβ)-Octahydro-7-[1-(hydroxymethyl)ethenyl]-1,8a-dimethylnaphthalen-4a(2H)-ol ■	$C_{15}H_{26}O_2$	238				0.89	0.61		
15	24.31	Elemol	$C_{15}H_{26}O$	222							1.71
16	24.58	(-)-7*β*H-Eudesmane-4*α*,11-diol◇	$C_{15}H_{28}O_2$	240			0.62				0.85

17	24.59	[3S-(3a,3β,5a)]-1,2,3,3a,4,5,6,7-Octahydro-.a,.a,3,8-tetramethyl-5-azulenemethanol□	$C_{15}H_{26}O$	222							0.64
18	25.25	Dihydrokaranone ■	$C_{15}H_{22}O$	218			0.82	1.04	1.95	0.54	
19	25.36	Valenca-1(10),8-dien-11-ol▪	$C_{15}H_{24}O$	220	1.35		0.94	0.94	4.98	1.08	1.04
20	25.65	α-Kessyl alcohol□	$C_{15}H_{26}O_2$	238							1.40
21	25.74	2,t-3-Dimethyl-r-2-(3-methyl-2-butenyl)-1-cyclohexanone■	$C_{13}H_{22}O$	194	0.61		1.63	4.65	1.05	0.94	
22	25.80	4-Hydroxyl-baimuxinol★	$C_{15}H_{26}O_3$	254					0.88		1.45
23	25.88	Baimuxinal ☆	$C_{15}H_{24}O_2$	236	0.64		3.02	2.50	4.09	2.80	0.89
24	26.26	Isobaimuxinol★	$C_{15}H_{26}O_2$	238						1.19	2.87
25	26.96	(-)-2α-Hydroxyguaia-1(10),11-dien-15-oic acid□	$C_{15}H_{22}O_3$	250	0.40	0.98	0.28	0.51		0.57	
26	27.35	Baimuxinol★	$C_{15}H_{26}O_2$	238					1.66		1.61
27	27.43	Qinanol D□	$C_{15}H_{26}O_3$	254							2.94
28	27.50	Qinanol A□	$C_{15}H_{26}O_2$	238							1.40
29	27.69	Qinanol C□	$C_{15}H_{26}O_3$	254							1.11
30	28.03	ent-4(15)-Eudesmen-11-ol-1-one◇	$C_{15}H_{26}O_2$	238	0.47						
31	28.48	7α-H-9(10)-ene-11,12-Epoxy-8-oxoeremophilane ■	$C_{15}H_{22}O_2$	234	0.50			0.85	0.75		
32	28.73	11-Hydroxy-valenc-1(l0)-en-2-one■	$C_{15}H_{24}O_2$	236			0.63		0.59	0.77	0.83
33	28.75	(−)-9-Hydroxy-selina-3,11-dien-14-al◇	$C_{15}H_{22}O_2$	234	0.68			1.22			
34	28.96	(−)-Guaia-1(10),11-dien-15-al□	$C_{15}H_{22}O$	218			0.47	1.01	1.38	0.29	

35	29.81	4-epi-15-Hydroxy- acorenone	$C_{15}H_{24}O_2$	236	0.60						
36	29.97	(+)-9-Hydroxy-selina-4,11-dien-14-al◇	$C_{15}H_{22}O_2$	234	0.52			3.24		0.62	
37	32.49	(−)-Guaia-1(10),11-diene-15,2-olide□	$C_{15}H_{20}O_2$	232		6.89	0.45	2.42	1.56		0.77
38	33.58	(+)-8,12-Dihydroxy-selina-4,11-dien-14-al◇	$C_{15}H_{22}O_3$	250					0.38		
39	33.79	Valerenol	$C_{15}H_{24}O$	220		0.80		0.77			0.45
40	33.92	2-[2-(2-Hydroxyphenyl)ethyl]chromone	$C_{17}H_{14}O_3$	266		0.84	0.72	0.92			
41	35.06	2-(2-Phenylethyl)chromone	$C_{17}H_{14}O_2$	250	19.45	25.69	42.64	34.61	40.86	32.33	24.69
42	38.62	2-[2-(4-Methoxyphenyl)ethyl] chromone	$C_{18}H_{16}O_3$	280	44.47	45.72	23.08	9.28	27.51	40.71	37.06
43	40.37	2-[2-(3-Methoxyphenyl)ethyl]chromone	$C_{18}H_{16}O_3$	280	6.56	3.27	4.3	0.89			
44	41.24	2-[2-(3-Methoxy-4-hydroxyphenyl)ethyl]chromone	$C_{18}H_{16}O_4$	296	2.21	0.79	1.67	0.77		0.41	1.26
45	41.28	2-[2-(2-Methoxyphenyl)ethyl]chromone	$C_{18}H_{16}O_3$	280	2.93	2.77		1.54			
46	41.33	6-Methoxy-2-(2-phenylethyl)chromone	$C_{18}H_{16}O_3$	280			3.38				
47	41.96	2-[2-(3-Hydroxy-4-methoxyphenyl)ethyl]chromone	$C_{18}H_{16}O_4$	296	6.26	4.74	2.12	0.48			
48	42.30	5,8-Dihydroxy-2-(2-phenylethyl)chromone	$C_{17}H_{14}O_4$	282				0.77			
49	43.24	8-Hydroxy-2-(2-phenylethyl)chromone	$C_{17}H_{14}O_3$	266	1.33	0.76		0.76			
50	43.29	6-Hydroxy-2-(2-phenylethyl)chromone	$C_{17}H_{14}O_3$	266				6.89			
51	45.03	6-Methoxy-2-[2-(3-methoxyphenyl)ethyl]chromone	$C_{19}H_{18}O_4$	310	0.88						
52	45.50	6-Methoxy-2-[2-(4-methoxyphenyl)ethyl]chromone	$C_{19}H_{18}O_4$	310	4.03	0.68		0.90			

53	45.52	6,7-Dimethoxy--2-(2-phenylethyl)chromone	$C_{19}H_{18}O_4$	310				1.33			
54	46.96	6-Hydroxy-2-[2-(4-methoxyphenyl)ethyl]chromone	$C_{18}H_{16}O_4$	296	0.50						
55	47.46	6-Methoxy-2-[2-(3-methoxy-4-hydroxylphenyl)ethyl] chromone	$C_{19}H_{18}O_5$	326	0.44						
56	48.16	6-Methoxy-2-[2-(3-hydroxyl-4-methoxyphenyl)ethyl] chromone	$C_{19}H_{18}O_5$	326	0.35						
		■：The relative content of eremophilane sesquiterpenes			2.46	1.03	4.83	11.23	11.26	3.33	2.72
		The number of eremophilane sesquiterpenes			3	2	5	8	8	4	3
		□：The relative content of guaiane sesquiterpenes			0.40	7.87	1.20	3.94	2.94	0.86	11.05
		The number of guaiane sesquiterpenes			1	2	3	3	2	2	8
		◇：The relative content of eudesmane sesquiterpenes			1.67	0	0.62	4.46	1.31	0.62	0.85
		The number ofeudesmane sesquiterpenes			3	0	1	2	2	1	1
		★：The relative content of agarofurans sesquiterpenes			0	0	0	0	2.54	1.19	6.37
		The number of agarofurans sesquiterpenes			0	0	0	0	2	1	4
		☆：The relative content of agarospirane sesquiterpenes			0.64	0	3.02	2.50	4.09	2.80	2.90
		The number of agarospirane sesquiterpenes			1	0	1	1	1	1	2
		The relative content of all sesquiterpenes			5.77	9.70	9.67	22.90	22.14	8.80	26.55
		The number of all sesquiterpenes			9	5	10	15	15	9	21
		The relative content of chromones			89.41	85.26	77.91	59.14	68.37	73.45	63.01
		The number of chromones			12	9	7	12	2	3	3
		The relative content of No.41 and No.42			63.92	71.41	65.72	43.89	68.37	73.04	61.75
		The relative content of other chromones			25.49	13.85	12.19	15.25	0	0.41	1.26
		The number of other chromones			10	7	5	10	0	1	1
		Total			95.18	94.96	87.58	82.04	90.51	91.08	92.19

Note: eudesmane sesquiterpene; □: guaiane sesquiterpene;☆: agarospirane sesquiterpene； ■: eremophilane sesquiterpene;★: agarofurans sesquiterpene.

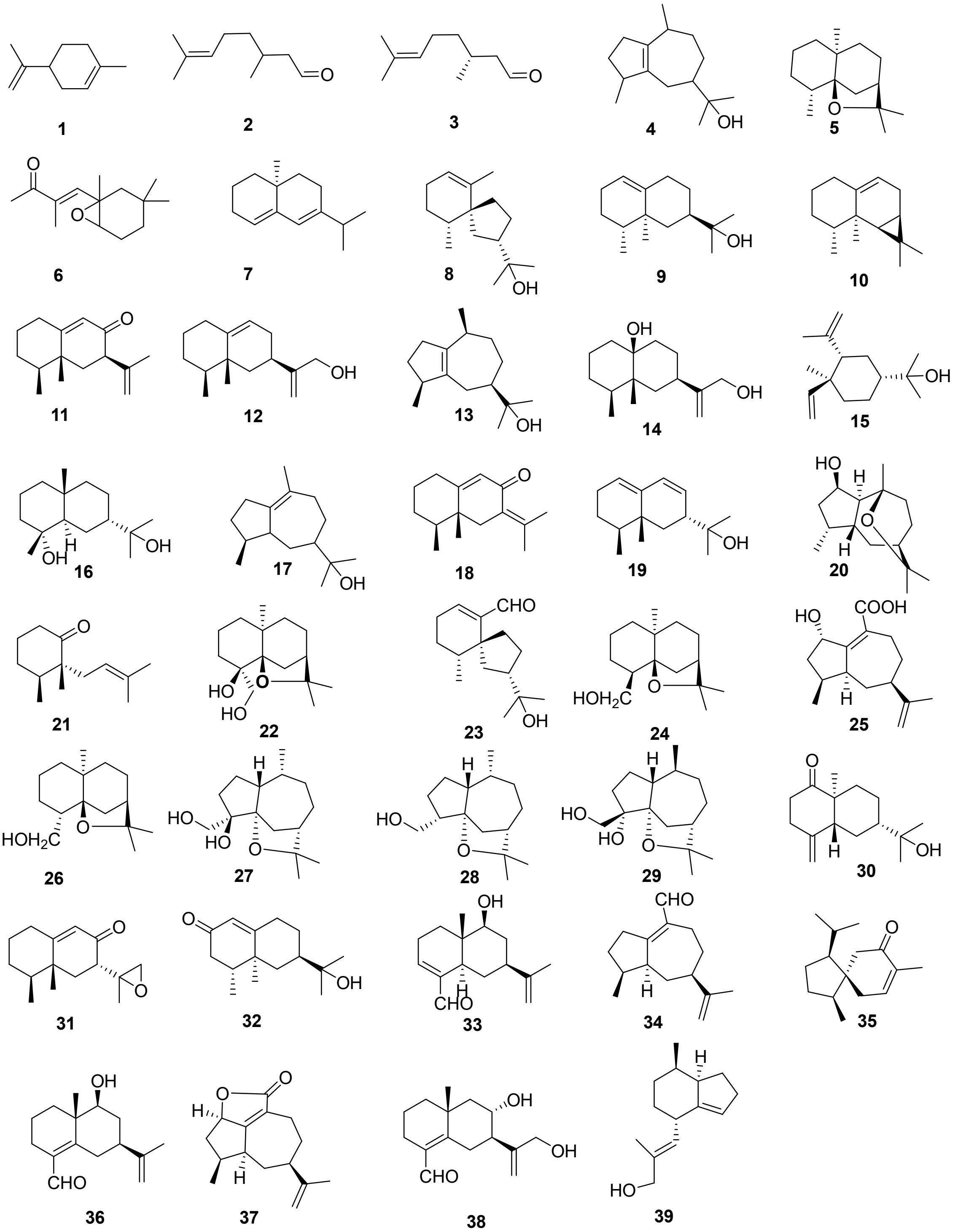

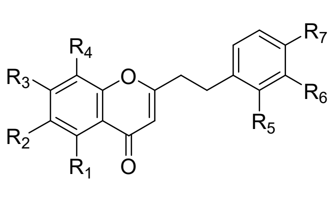

40:R_5=OH,R_1~R_4,R_6,R_7=H
41:R_1~R_7=H
42:R_7=OCH_3,R_1-R_6=H
43:R_6=OCH_3,R_1-R_5,R_7=H
44:R_6=OCH_3,R_7=OH,R_1~R_5=H
45:R_5=OCH_3,R_1~R_4,R_6,R_7=H
46:R_2=OCH_3,R_1,R_3~R_7=H
47:R_7=OCH_3,R_6=OH,R_1-R_5=H

48:R_1=R_4=OH,R_2,R_3,R_5~R_7=H
49:R_4=OH,R_1-R_3,R_5-R_7=H
50:R_2=OH,R_1,R_3-R_7=H
51:R_2=R_6=OCH_3,R_1,R_3-R_5,R_7=H
52:R_2=R_7=OCH_3,R_1,R_3-R_6=H
53:R_2=R_3=OCH_3,R_1,R_4-R_7=H
54:R_2=OH,R_7=OCH_3,R_1,R_3-R_6=H
55:R_2=R_6=OCH_3,R_7=OH,R_1,R_3-R_5=H
56:R_2=R_7=OCH_3,R_6=OH,R_1,R_3-R_5=H

Figure 4. Structures of compounds identified in wild harvested Qi-Nan agarwood by GC-MS.
